# Case of Spontaneous Version

**Published:** 1854-07

**Authors:** 


*Case of Spontaneous Version.* By John W. Tripe, M. D.

Cases of this kind are always interesting, and this case more than ordinarily so, because there is reason to agree with Dr. Tripe, in believing that it was originally one of back-presentation. The fact, that both shoulders were engaged in the pelvic cavity, and that an arm had been brought down in the belief that it was a leg, gives support to this idea. Dr. Tripe writes:—

A person came to me, informing me that a woman, who had been in labor since Monday last, required immediate assistance. On my arrival, I found the patient suffering with sharp and quick labor-pains, an intensely-flushed face, furred tongue, with a dry, brown tip, and rather quick pulse. I was also informed, that the midwife who had been in attendance had ruptured the membranes on Monday, (to-day being Thursday,) labor having commenced on the Sunday evening. It had been considered a breech-case, until one of the hands was protruded through the os externum. On looking at the hand, I found the cuticle peeled off, the child having evidently been dead for some days. On passing my hand to the presenting part, I was struck by finding both shoulders engaged in the pelvic cavity, so that I could pass my finger over the left shoulder, which was highest, into, the corresponding axilla, although the right hand was external. Both scapulæ could readily be felt, their bases being quite close together, and, with some difficulty, the extremely dense and elongated neck could be traced upward in a direction between the symphysis pubis and right acetabulum, the occiput resting on the linea ileo-pectinea, above the foramen thyroideum; the shoulders being placed obliquely, the right near the left sacro-iliac synchondrosis, and the left near the right acetabulum. On inquiry, I found that the arm had been pulled down, after which, the pains being strong, both shoulders had been jammed down as I found them. I then introduced my left hand into the uterus, for the purpose of turning, and, after great difficulty, succeeded in reaching the feet, which were situated just above the head, facing the mother's abdomen, the back being obliquely toward the spine. On using the necessary power to effect version, I found that it was chiefly expended on the head, which was then more tightly brought down in the linea-ileo pectinea, and that no power which I dared to use, for fear of injuring the bladder, would effect the object sought. The patient was so ungovernable, that I was compelled to desist, after a short trial. After a short interval, I attempted to reach the feet by passing my right hand over the head, and thus pushing it away from its position, but was again foiled by the unruliness of my patient. I then sent for a sharp hook to perform decapitation, and, in the interval, gently pushed up the left shoulder, the pains having gone off, so that the right arm, shoulder, and side, were made the lowest parts. I did this in the hope that, the pelvis being large, and the child small (eight months,) and decomposed, spontaneous version might take place. On the return of the messenger the patient refused to allow me to touch her, and became very violent, but took some brandy and water. After the lapse of nearly half an hour, the pains returned, and, on the occurrence of the second, which was a strong one, she cried out, "The child is coming!" when I was just in time to receive the breech in my hand, the child being completely expelled by the same pain, the head being the part which came out last. I regret very much not having had an opportunity of tracing the whole process, but the patient would not allow me to make an examination. After the child was born, a rather extensive flooding occurred, from the placenta being slightly adherent, but it ceased on the removal of this organ.—*Medical Times & Gazette.*

